# Comparison of Subjective Workout Intensities between Aquatic and Land-based Running in Healthy Young Males: A Pilot Study

**DOI:** 10.3390/medicina56040151

**Published:** 2020-03-28

**Authors:** Chang-Hyung Lee, Jun Hwan Choi, Soo-Yeon Kim

**Affiliations:** 1 Rehabilitation Medicine, Pusan National University School of Medicine and Research Institute for Convergence of Biomedical Science and Technology, Pusan National University Yangsan Hospital, Yangsan 50612, Korea; aarondoctor@gmail.com; 2 Department of Rehabilitation Medicine, Jeju National University Hospital, Jeju National University School of Medicine, Jeju 63241, Korea; miraerojh0728@gmail.com

**Keywords:** aquatic exercise, cardiorespiratory response, shallow-water running, rating of perceived exertion

## Abstract

*Background and objectives:* Aquatic exercises have demonstrated several advantages over land-based exercise, but only a few studies have compared the workout intensities and efficiencies in a stage-specific manner. This study aimed to investigate workout intensity during aquatic and land-based running, based on the rating of perceived exertion (RPE). *Materials and Methods:* Twenty healthy young male subjects underwent a land-based running test (LRT) and an aquatic running test (ART), in the form of a cardiopulmonary exercise treadmill test and a shallow-water running test. The seven stages of the ART were composed of 3 minutes each of the Bruce protocol performed during the LRT. In the ART, the participants were instructed to run in a swimming pool with matching RPE to that obtained at each stage of the LRT. *Results:* Heart rate (HR) during both LRT and ART exhibited a linear relationship (*r* = 0.997 and 0.996, respectively; *p* < 0.001). During the initial and middle period, HR was higher in the ART than in the LRT. However, in the final period, HR was higher in the LRT than in the ART. *Conclusions:* In aquatic exercises based on the RPE obtained from the LRT, HR exhibited a linear relationship in both the ART and the LRT. The ART appears to increase cardiac loading more efficiently in the initial period and does not increase cardiac loading abruptly at a later period. Although there is no precise, objective, controlled parameter to compare the ART and the LRT, the RPE may be used as a convenient measurement for workout intensity in aquatic running.

## 1. Introduction

Aquatic exercises are popular activities in the context of fitness, therapy, and rehabilitation [1]. Aquatic exercises have several advantages over land-based exercises; they lower risks of injury by reducing musculoskeletal loading [2], provide a sense of comfort, safety and psychological benefit due to freedom from concerns of falling down [3], enable aerobic and resistance exercises to be combined by utilizing the resistance of water [4,5], and, finally, aid in weight reduction by increasing energy expenditure [6]. Accordingly, aquatic exercises have recently been prescribed for cardiorespiratory fitness, injured athletes, and the elderly [3,7,8].

Aquatic running methods include deep-water running (DWR) and shallow-water running (SWR) [9]. During DWR, participants run while fitted with a buoyant belt or vest across a swimming pool. DWR differs from land running in terms of kinematics and lower limb muscle recruitment [10], due to the absence of a ground support phase and the involvement of water resistance. On the other hand, during SWR, participants run without any buoyant device and are typically immersed between the waist and xiphoid process. SWR differs from DWR in terms of the presence of ground reaction force, which is lower compared to land running and depends on the depth of immersion. Regardless of water depth, aquatic exercise has been suggested as a good therapeutic method, as it decreases axial loading on the musculoskeletal structure [1,11]. One can exercise safely in water without inflicting unnecessary load on the musculoskeletal structure due to weight [7].

However, the efficiency of aquatic exercise has not been clearly demonstrated in previous studies. A majority of the studies on this topic have demonstrated that aquatic running is characterized by lower workout intensity than land-based treadmill running, as indicated by lower cardiorespiratory responses such as peak oxygen consumption (VO_2_, mL/kg/min) and heart rate [3,12,13]. This observation can be explained by several factors such as the absence of, or a low, ground reaction force [14], different water temperatures [15,16], no objective measurement method such as self-selected exercise intensity [2], and lack of familiarity with aquatic activities [17]. Unlike other exercises, methods for conventional exercise prescription and the measurement of workout intensity for aquatic exercises are rather limited. The most frequently used method of measuring results from aquatic exercises depends on ratings of perceived exertion (RPE) and heart rate (HR, bpm) [1,5,18]. The former is generally rated on the Borg CR10 Scale [19] in clinics and could be defined as a subjective rating of the intensity of a specific exercise. Physicians usually prescribe water-based exercises using an appropriate intensity scale of 0 to 10, in accordance with the workout capacity of the patients. However, the measurement of the precise workout intensity in a controlled manner could enable an objective comparison between aquatic and land-based exercises.

Although there has been a growth in interest in the use of aquatic exercises for therapy or training, only a few studies have examined means of measuring or controlling the workout intensities associated with aquatic exercises. On the basis of their findings, and on its strong correlation with heart rate during aquatic running, Wilder et al. suggested a cadence that provided subjects with a rhythm for regular limb movements as a measure of the intensity of aquatic running [18]. In a study on the exercise intensities of water-based activities, Raffaelli et al. demonstrated that various workout intensities can be obtained by changing movement frequencies and exercise types, such as jumping, running, and kicking [20]. However, previous studies on aquatic exercises with buoyancy devices or anaerobic exercises showed that these conditions could influence workout intensities. In addition to the simple comparison of total workout intensity, there are no previous studies demonstrating the differences in terms of intensity increment. Apparently, the above-mentioned results did not provide a precise comparison between aquatic and land-based exercise. To compare the precise workout intensity between the two methods, the standard protocol for exercise method and stage evaluation should be compared using the same standard measurement methods. In addition, although aquatic exercises have demonstrated several advantages over land-based exercises, only a few studies have compared the workout intensities and efficiencies in a stage-specific manner [3,7,13]. In previous studies of aquatic exercises, aquatic treadmills were used to control walking speed and workout intensities (HR, VO_2_, and RPE) for comparison with land-based running. However, measuring cardiorespiratory responses, such as VO_2,_, during running in a swimming pool in clinical practice is challenging. The aim of this study was to assess whether subjective perception reflects exercise workout intensity through a comparison between aquatic and land-based running based on the RPE.

## 2. Materials and Methods

Twenty healthy young males with no history of musculoskeletal injury and not undergoing any pharmacological therapy were recruited for this study. To rule out age and sex differences, we recruited only young males regardless of prior experience in aquatic running. To determine inclusion and exclusion criteria, the participants were asked to respond to a questionnaire. The inclusion criteria were as follows: (1) age ranging from 20 to 39 years (mean, 26.0 ± 7.3 years); (2) stature over 160 cm (mean, 172.1 ± 3.7 cm); (3) body weight ranging from 60 to 75 kg (mean, 66.2 ± 7.3 kg); and (4) body mass index (BMI) ranging from 20 to 26 kg/m^2^ (mean, 22.3 ± 2.7 kg/m^2^). All subjects received written and oral instructions before the test and provided informed consent. This study was approved on 14 Jan 2014 by the institutional review board (IRB 05-2012-018).

### 2.1. Experimental Setting for Aquatic Running Test (ART)

We suggested an ART protocol for subjective exercise intensity that can be conveniently used in clinical practice. The water level was adjusted to be between the xiphoid process and the jugular notch. No buoyancy device was utilized to prevent any possible influence on the workout intensities. ART was performed in the form of SWR to gain optimal exercise efficiencies with relatively moderate ground reaction force and much lower physical burden compared to the land-based running test (LRT). The workout intensities of LRT were assessed by measuring cardiorespiratory responses such as HR, VO_2_, and RPE, which provide objective and subjective measures of exercise intensity. Additionally, the workout intensities of ART were assessed by measuring HR and RPE.

### 2.2. Aquatic Running and Land-Based Running Procedures

**Figure 1:**
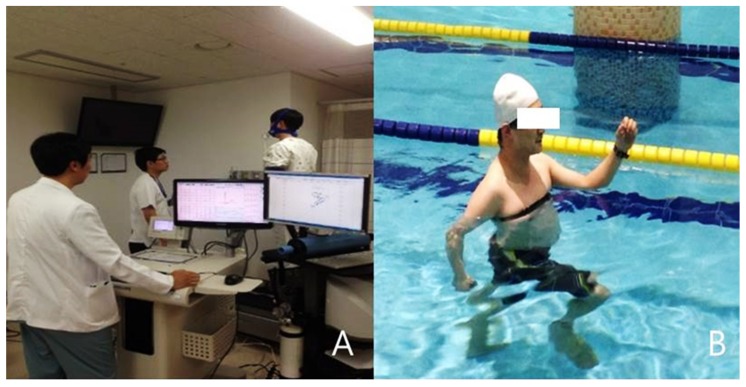
The land-based running test (LRT) (**A**) and the aquatic running test (ART) (**B**). LRT was performed on an incline-adjustable treadmill with continuous vital sign and electrocardiographic monitoring. ART was performed in a swimming pool with a water level between the xiphoid-process and the jugular notch, with monitoring of heart rate (HR) using a water-resistant chest-strap transmitter.

All 20 subjects participated in two running tests. One was a land-based running test in the form of a cardiopulmonary exercise treadmill test (CPET), and the other was an aquatic running test (Figure 1).

The LRT was performed before the ART and participants had a rest interval between the two testing sessions of at least 72 hours to maximize performance in each protocol.

Each running test included a warm-up exercise for 5 minutes. The peak VO_2_, HR, and RPE during LRT were measured during the test. LRT was performed on a calibrated, incline-adjustable treadmill (STEX 8100T, TaeHa, Korea) with real-time recording 12-channel electrocardiographic monitoring (Philips Health Care 3000 Minuteman Rd., Andover, MA, USA) and vital sign monitoring based on the Bruce protocol. The Bruce protocol is a standard test in cardiology and comprises multiple exercise stages that each last 3 minutes. At each stage, the gradient and speed of the treadmill are elevated to increase work output, called METs (metabolic equivalent of task). Stage 1 of the Bruce protocol is performed at 1.7 miles per hour and at a 10% gradient. VO_2_ was determined by analyzing expired air through a breath-by-breath method using a portable telemetric system (Ultima Series™ metabolic stress-testing system, MGC Diagnostics, Saint Paul, Minnesota). For LRT, all the participants were instructed to increase their workout intensity on the treadmill test until the achievement of submaximal threshold (80% of maximal heart rate or an RPE of 8~9). The exercise test was terminated on the participant’s request or according to the guidelines of the American College of Sports Medicine [20]. RPE was assessed using a numerical rating scale, the Borg CR10 Scale (0–10).

ART was performed in a swimming pool, while heart rate was monitored using a water-resistant chest-strap transmitter (Polar T34, Polar Electro, Inc, Kempele, Oulu, Finland), with the water level reaching between the xiphoid process and the jugular notch (water temperature, 31 °C). During ART, all participants were instructed to run by moving their arm back and forth without swimming while their legs continued to run in the pool for 3 minutes in each stage, similar to the Bruce protocol performed in the LRT. To gradually and constantly increase the workout intensity in water, we defined the workout intensity as HR of ART as the matching RPE that was obtained at each stage of LRT. After acquiring all seven stages, the stages were classified as follows: initial (stages 1 and 2), middle (stages 3 to 5), and final (stages 6 and 7). During ART, heart rate was measured at rest and at each stage.

### 2.3. Statistical Analysis

Kolmogorov–Smirnov verification was used to prove the normality of the data, which were found to be not normally distributed. A Wilcoxon signed-rank test was used to assess the difference in heart rate between the ART and the LRT at each stage. Spearman’s correlation was used to analyze the stage-associated differences. Statistical analyses were performed using SPSS version 21.0 for Windows (SPSS Inc., Chicago, IL, USA). Statistical significance was accepted for *p* values < 0.05 and <0.001 respectively.

## 3. Results

Twenty male subjects completed LRT and ART on separate days. At the end of each exercise, the peak VO_2_, HR, and RPE in LRT were 43.8 ± 3.9 mL/kg/min, 179.5 ± 9.7 bpm, and 8.70 ± 0.82, respectively. The peak HR for ART at stage 7 were 172.2 ± 4.7 bpm. The final workload for the LRT (which is equivalent to stage 7 of ART) was at a speed of 6.0 mph and at an inclination of 22% (Table 1).

**Figure 2:**
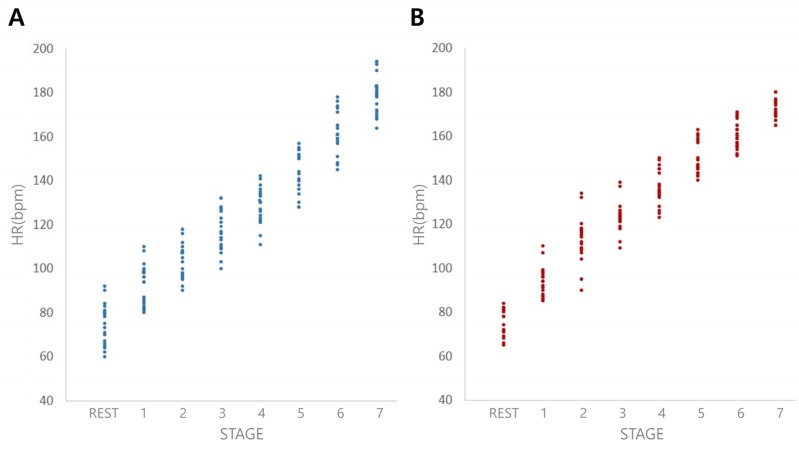
Increase in heart rate (HR) observed during the seven stages of the land-based running test (LRT) (**A**) and the aquatic running test (ART) (**B**) from the rest state. Linear relationship between HR and the seven stages in LRT (**A**) and ART (**B**) (*r* = 0.997 and 0.996, respectively, *p* < 0.001, Spearman’s correlation test); bpm, beats per minute.

HR during both LRT and ART exhibited a linear relationship (*r* = 0.997 and 0.996, respectively; *p* < 0.001) (Figure 2). During the initial and middle period, HR was higher in ART than LRT; however, in the final period, HR was higher in LRT than ART. Statistically significant differences were observed between LRT and ART for HR during stages 2, 3 and 7 (*p* < 0.05) (Table 2).

## 4. Discussion

To prescribe exercise intensity or predict the resulting outcome, precise and controlled exercise protocols should be prepared. Although the beneficial effects of aquatic exercises have been widely accepted, the workout intensity should be considered in exercise prescription for therapy or rehabilitation to obtain optimal positive effects while avoiding possible injury [7,21].

Measurements of total calorie loss or VO2max are useful for acquiring an objective measure of workout intensity. However, in the absence of appropriate respiratory aquatic analysis equipment, obtaining such data is challenging, especially in a swimming-pool-based exercise. Theoretically, subjective parameters used to measure workout intensity have an advantage over objective parameters in reflecting the actual ’perceived intensity’ of individuals. Although the same protocol for workout intensity is performed in water, the output (HR in this study) could be inconsistent because exercise output could be strongly influenced by individual physical characteristics (e.g., running performance in water) [10] and environmental factors (e.g., water temperature, relative depth of water) [16,22]. In this study, we measured the subjective RPE value, and the gradual increase in its intensity in accordance with each stage of the LRT. Due to the viscosity and consistency of water, cadence should not be used as the standard measurement method as speed increases. Difficulty perceptions in the ART increased with increasing intensity, which corresponded to the RPE in the LRT. HR in both the LRT and the ART with matching RPE exhibited a linear relationship (*r* = 0.997 and 0.996, respectively; *p* < 0.001), and there were no statistical differences, except in stages 2, 3, and 7. Although there is no precise objective parameter compared between the ART and the LRT, the RPE may potentially provide a fundamental, convenient, and meaningful parameter in aqua that reflects an “actual perceived workout intensity” measure in individuals.

In the present study, HR as exercise workout of ART and LRT were assessed and compared (Table 2). The plots shown in Figure 2 show an increase in HR and during the three defined test periods for both ART and LRT. Initially, ART exhibited higher HR values than LRT during the initial and middle periods. Conversely, HR was lower in ART than LRT during the final period (stage 5 to 6). Theoretically, the ground reaction force due to counter dragging force can be controlled in our method, thus enabling exercise intensity to be increased gradually during the course of the test. The maximum kinetic effort was observed during the early stage of the ART, presumably due to the higher metabolic energy required to overcome the drag force on lower limbs to initiate aquatic running [14], and at this stage HRs were higher for the ART than in the LRT. Secondly, the mechanical counterforce corresponding to the water depth should be considered. If a person moves more vigorously in water, more counterforce is imposed, thus leading to an increase in HR and RPE. However, the kinetic effort could not increase exponentially, due to the enhanced counterforce at a later period. Despite the greater effort at a later period, the actual increase in cardiac loading turned out to be small compared to LRT.

It was hypothesized that precise measurement of the workout intensity and effectiveness of aquatic exercise in a stage-specific manner could allow objective comparisons, thus enabling prescription of aquatic exercises in a more objective and safe manner in the clinical setting. In the present study, comparison of the aquatic workout intensity with LRT using RPE as a parameter provided subjective data which could be useful in clinical practice. A higher HR in ART compared to LRT was observed until the middle exercise period, therefore suggesting a higher magnitude of enhancement of workout intensities during the initial and middle period. Aquatic running, a higher-intensity exercise workout that is less physically burdening than land-based running, is likely to be especially advantageous for elderly, obese, and severely ill patients. Consequently, aquatic exercise can be recommended over land exercise for patients with a deconditioned or weakened physical status (for example, patients suffering from osteoarthritis or rheumatoid arthritis) [23]. Furthermore, the lower HR during the final period of ART relative to LRT provides a safer exercise window. Considering these aspects, ART could be safely recommended in clinical settings, as it is also not as demanding as LRT with regard to its physical burden, especially in patients with a low cardiac ejection fraction below 55% (e.g. heart failure) [24].

There are several possible explanations for the observed workout differences. The physiological adaptation to water should be considered. In a cross-sectional study, it was demonstrated that subjects who participated in a session of aquatic exercise achieved acute adaptation. For example, as pool water temperature decreased, HR increased rapidly in response to sympathetic nervous system stimulation [16,22]. The increase in peripheral resistance with vasoconstriction is due to the blood being redirected from the periphery to maintain core temperature [25]. It has been shown that immersion at neutral temperature (32 °C) lowers HR by 15%, but immersion in cold water (14 °C) increases HR by 5% [16]. In the present study, the swimming pool temperature was maintained at 31 °C; therefore, we believe that the effect of water temperature on HR was minimal during the early stage of exercise. On the other hand, RPE has been reported to decrease with the depth of immersion [26], and, in another study, high RPE appeared to be positively related to ground reaction force, the amount of drag force on lower limbs [14], and changes in the neuromuscular patterns of active muscles [27]. As ground reaction forces are always lower during shallow water running (SWR) than during land running, musculoskeletal burden and the risk of injury could be reduced during SWR.

However, previous studies have demonstrated a decrease in HR with an increase in the water depth [17,26], and that the decline in HR is associated with the influence of hydrostatic pressure and buoyancy on the stroke volume of heart and consequent alteration in blood distribution in the body [18]. In the present study, the water level was adjusted to a level between the xiphoid process and the jugular notch. This depth was more than the specific level used for SWR but shallower than that used for DWR. Consequently, it was hypothesized that the lower HR observed during the later period of ART compared to LRT was due to the water depth employed in this study.

Generally, the water depth in swimming pools is usually at adult chest height, and a similar level was employed in the present study. Apparently, it is hypothesized that our findings are meaningful, based on the observation that the workout intensities of aquatic exercises reflect real situations, and are applicable to aquatic exercises in clinical practice.

There are several results of clinical importance in our study. As there was previously no exact guideline on intensity in aquatic therapy, these findings might suggest a possible guideline for aquatic exercise. Initially, ART can increase the heart rate efficiently, which appears to be a good choice for patients in a deconditioned state or unable to walk. Meanwhile HR during the ART rises slower than during the LRT at later stages. This relatively safe ‘window period’ could be safely suggested in patients with cardiac problems. In general, heart rate increases linearly in relation to RPE. However, the heart rate did not increase linearly in later stages of ART. This might reflect the increasing viscosity and resistance with the increase in running effort, increasing demand for endurance and muscle fatigue in the water, and hence increasing thermal loss in ART.

There are several limitations of this study. First, all study subjects were young male adults; thus, our results are applicable primarily to this population and not to female subjects, the elderly, or patients with cardiac problems. Second, we only considered the partial influence of cardiorespiratory response on the workout intensity of the ART by measuring HR with controlled RPE, but not VO_2_, because of the absence of an appropriate respiratory gas analysis system at the swimming pool. In future studies, it will be necessary to confirm whether RPE, a subjective parameter, is a meaningful indicator that can reflect and objectively measure exercise intensity in aquatic exercises in various conditions.

## 5. Conclusions

This study demonstrates the workout intensities of aquatic and land-based running based on RPE. The workout difficulty perceptions in aquatic running increased with increasing intensity, which corresponded to RPE in the land-based running test. Although there is no precise comparison of controlled cardiorespiratory measurement to compare aquatic and land-based running, RPE may be used as a meaningful measurement for workout intensity in aquatic running.

## Figures and Tables

**Table 1:** Heart rate (HR), rating of perceived exertion (RPE), and oxygen consumption (VO_2_) measured at rest state and during the seven stages of the land-based running test (LRT).

Stage	HR (bpm)	RPE	VO_2_ (mL/kg/min)
Rest	74.9 ± 9.6	0	3.3 ± 0.8
Stage 1	91.8 ± 10.4	0.3 ± 0.2	8.9 ± 1.0
Stage 2	103.8 ± 8.5	1.2 ± 0.6	13.2 ± 1.3
Stage 3	117.3 ± 9.9	2.5 ± 0.7	18.0 ± 1.4
Stage 4	128.5 ± 9.2	3.7 ± 0.6	24.1 ± 2.4
Stage 5	144.2 ± 10.1	5.1 ± 0.8	30.3 ± 3.9
Stage 6	161.4 ± 11.2	6.8 ± 1.1	36.3 ± 4.3
Stage 7	179.5 ± 9.7	8.7 ± 0.8	43.8 ± 3.9

RPE scores were obtained using the Borg CR10 Scale (0 to 10); Values are presented as mean ± standard deviation (SD); bpm, beats per minute.

**Table 2:** Comparison of heart rate (HR) between land-based running test (LRT) and aqua-based running test (ART) according to stages.

Stage	HR (bpm)				*Z*-score	*p* Value
	LRT		ART			
	(Mean ± SD)	(Median)	(Mean ± SD)	(Median)		
Rest	74.9 ± 9.6	77.5	76.1 ±6.6	78.0	−1.029	0.304
Stage 1	91.8 ± 10.4	91.5	93.9 ± 7.7	93.5	−1.188	0.235
Stage 2	103.8 ± 8.5	106.0	112.7 ± 11.1	113.0	−2.298	0.022 *
Stage 3	117.3 ± 9.9	116.5	123.4 ± 7.5	124.0	−1.989	0.047 *
Stage 4	128.5 ± 9.2	129.0	136.4 ± 8.4	135.5	−1.888	0.059
Stage 5	144.2 ± 10.1	144.0	149.4 ± 7.6	145.5	−1.071	0.284
Stage 6	161.4 ± 11.2	161.0	160.4 ± 6.3	160.0	−0.358	0.721
Stage 7	179.5 ± 9.7	179.5	172.2 ± 4.7	172.5	−2.052	0.040 *

* p < 0.05, Wilcoxon signed-rank test; bpm, beats per minute; Z-score using the normal approximation to the binomial distribution.
